# Pseudotyped αvβ6 integrin-targeted adenovirus vectors for ovarian cancer therapies

**DOI:** 10.18632/oncotarget.8545

**Published:** 2016-04-01

**Authors:** Hanni Uusi-Kerttula, James Davies, Lynda Coughlan, Sarah Hulin-Curtis, Rachel Jones, Louise Hanna, John D. Chester, Alan L. Parker

**Affiliations:** ^1^ Department of Cancer and Genetics, School of Medicine, Cardiff University, Cardiff CF14 4XN, UK; ^2^ Nuffield Department of Medicine, The Jenner Institute, University of Oxford, Oxford OX3 7DQ, UK; ^3^ Velindre Cancer Centre, Cardiff CF14 2TL, UK

**Keywords:** adenovirus, re-targeting, neutralizing antibody, ovarian cancer, αvβ6 integrin

## Abstract

Encouraging results from recent clinical trials are revitalizing the field of oncolytic virotherapies. Human adenovirus type 5 (HAdV-C5/Ad5) is a common vector for its ease of manipulation, high production titers and capacity to transduce multiple cell types. However, effective clinical applications are hindered by poor tumor-selectivity and vector neutralization. We generated Ad5/kn48 by pseudotyping Ad5 with the fiber knob domain from the less seroprevalent HAdV-D48 (Ad48). The vector was shown to utilize coxsackie and adenovirus receptor (CAR) but not CD46 for cell entry. A 20-amino acid peptide NAVPNLRGDLQVLAQKVART (A20) was inserted into the Ad5. Luc HI loop (Ad5.HI.A20) and Ad5/kn48 DG loop (Ad5/kn48.DG.A20) to target a prognostic cancer cell marker, αvβ6 integrin. Relative to the Ad5.Luc parent vector, Ad5.HI.A20, Ad5.KO1.HI.A20 (KO1, ablated CAR-binding) and Ad5/kn48.DG.A20 showed ∼ 160-, 270- and 180-fold increased transduction in BT-20 breast carcinoma cells (αvβ6^high^). Primary human epithelial ovarian cancer (EOC) cultures derived from clinical ascites provided a useful *ex vivo* model for intraperitoneal virotherapy. Ad5.HI.A20, Ad5.KO1.HI.A20 and Ad5/kn48.DG.A20 transduction was ∼ 70-, 60- and 16-fold increased relative to Ad5.Luc in EOC cells (αvβ6^high^), respectively. A20 vectors transduced EOC cells at up to ∼ 950-fold higher efficiency in the presence of neutralizing ovarian ascites, as compared to Ad5.Luc. Efficient transduction and enhanced cancer-selectivity via a non-native αvβ6-mediated route was demonstrated, even in the presence of pre-existing anti-Ad5 immunity. Consequently, αvβ6-targeted Ad vectors may represent a promising platform for local intraperitoneal treatment of ovarian cancer metastases.

## INTRODUCTION

Ovarian cancer remains the sixth most common cancer in women, accounting for approximately 150 000 annual deaths worldwide [1]. Many patients present with advanced disease and despite good initial responses to systemic chemotherapy, aggressive, platinum-resistant tumors rapidly develop. Despite the introduction of new drugs, there has been little improvement in overall survival over the past 20 years. An urgent need for new treatments to combat recurrent disease persists. Oncolytic viruses, engineered to selectively infect and lyse cancer cells, hold great promise in this setting and can be coupled with concurrent expression of high levels of therapeutic transgenes. Achieving local high-level replication, amplification and tumor cell lysis, together with the stimulation of anti-tumor immune responses, offers selectivity and power unmatched by other anti-cancer therapies.

Human adenoviruses (HAdV/Ad), particularly species C type 5 (HAdV-C5/Ad5), have found favor as agents for virotherapy. They efficiently transduce a wide range of dividing or non-dividing cell types, are easy to genetically engineer and can be propagated to extremely high titers (> 10^13^ viral particles/mL, vp/mL). The mechanisms underpinning Ad5 cellular uptake and tropism *in vitro* are well-studied and clearly understood (reviewed in [2]). Cellular uptake occurs via binding of the Ad5 fiber protein to coxsackie and adenovirus receptor (CAR) [3]. Internalization involves a secondary, endocytosis-stimulating binding between the Ad5 penton base protein – via the conserved Arg-Gly-Asp (RGD) motif [4] – and αvβ3/5 integrins [5] on the host cell surface. CAR is ubiquitously expressed across human tissues, including erythrocytes [6–8] and on a variety of tumor cells, although a number of reports have associated tumor progression with loss of CAR expression [9, 10]. As virotherapy based on CAR-utilizing vectors may be suboptimal for efficient tumor-targeting, evaluation of less common Ad types with alternative receptor tropisms is warranted.

Systemic cancer virotherapy using Ad5-based vectors is hampered by binding to host blood cells, pre-existing anti-viral neutralizing antibodies (nAbs) and other proteins in the circulating blood. This results in rapid vector elimination and/or toxic adverse effects (reviewed in [2]). A recent epidemiological study with approximately 1900 participants from eight geographical locations reported the prevalence of anti-Ad5 nAbs to be highest in Thailand (94 %), with overall prevalence of anti-Ad5 nAbs being 85 % and lowest for HAdV-D36 (46 %) [11]. Species D Ads are appealing candidates as they have low seroprevalence, including in North and South American, sub-Saharan African and Southeast Asian populations [12, 13]. In this study Ad5 was pseudotyped with a fiber knob domain from HAdV-D48 (Ad48), generating a novel vector Ad5/kn48. The receptor usage of this vector was evaluated via *in vitro* competitive inhibition assays.

The limitations encountered with systemic delivery can be mitigated by local intratumoral or -cavity delivery of virotherapies. Therefore, we and others [14, 15] are developing viral vectors suitable for local intraperitoneal (i.p.) treatment of advanced ovarian cancer. The build-up of malignant ovarian ascites is an indicator of peritoneal metastases and poor prognosis. Ovarian ascites has a complex composition of various cell types and soluble proteins (reviewed in [16]), including high levels of anti-Ad5 nAbs that inactivate Ad5-based therapeutic vectors [17–20]. The fiber has been suggested to be the primary target for nAbs residing in ovarian ascites [17]. We therefore reasoned that evasion of pre-existing humoral anti-viral immunity in ovarian cancer patients might be facilitated by modifying our fiber-pseudotyped vector Ad5/kn48.

The epithelial-specific αvβ6 integrin is absent in healthy adult tissues [21, 22] but over-expressed in various cancer types, including ovarian cancer [23, 24]. Importantly, up-regulation of αvβ6 integrin has been suggested to correlate with disease progression [22, 25, 26]. We proposed to enhance cancer-selectivity using a previously-described 20-amino acid (aa) peptide, NAVPNLRGDLQVLAQKVART (A20) from foot-and-mouth disease virus (FMDV) VP1 capsid protein with native affinity to αvβ6 integrin [27]. A20 was genetically engineered into the HI loop of the Ad5 fiber knob domain (Ad5.HI.A20) [28], in CAR-binding ablated KO1 background [29] (Ad5.KO1.A20), and into the DG loop in the novel Ad5/kn48 vector (Ad5/kn48.DG.A20). The A20 viruses were assessed for transduction efficiency in αvβ6-expressing cancer cell lines. Ovarian ascites is a valuable source of primary epithelial ovarian cancer (EOC) cells that can be cultured [30, 31] for *ex vivo* evaluation of novel virotherapies [16]. Freshly-isolated clinical ovarian ascites-derived EOC cells from two donors were assessed with a view to designing improved oncolytic Ad vectors for i.p. treatment of advanced ovarian cancer. The combination of Ad5 capsid pseudotyping and αvβ6-targeting presents a promising personalized medicine approach for local vector delivery.

## RESULTS

### Recombinant vectors

**Figure 1:**
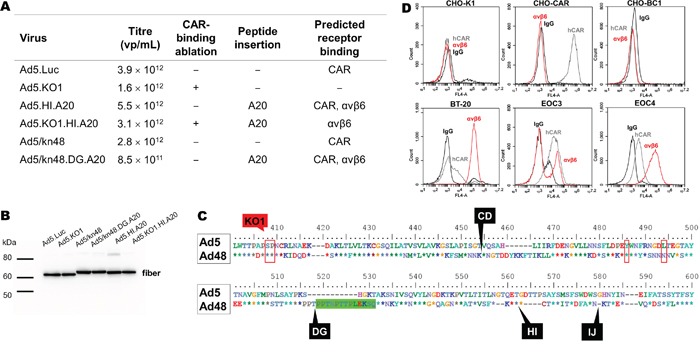
Generated vectors, cell line and primary epithelial ovarian cancer (EOC) cell phenotyping **A.** Vectors +/− αvβ6-targeting peptide (A20) in different knob loops (+/− CAR binding) were generated by AdZ recombineering [33], propagated into high titers and titrated by microBCA assay (1 μg = 4 × 10^9^ virus particles (vp)) [67]. **B.** Fiber integrity was confirmed on a Western blot using anti-Ad fiber antibody 4D2 (1:2000) with binding to residues (aa 1–17) outside those subject to modification; 1 × 10^10^ vp/lane. **C.** Ad5 and Ad48 fiber knob alignment: red boxes, CAR-binding sites [3]; green box, 13-aa deletion; black labels, A20 insertion sites/knob loops. **D.** Receptor expression profile of Chinese Hamster Ovary hamster ovarian (CHO-K1), CHO expressing CAR (CHO-CAR), CHO expressing CD46 (CHO-BC1), breast carcinoma (BT-20) cell lines and primary EOC cells from patients 3 and 4 were assessed by flow cytometry (normal mouse IgG, sc-2025; anti-CAR, Rcmb; anti-αvβ6, 10D5) and data analyzed on BD Accuri C6 software. CAR, human coxsackie and adenovirus receptor; KO1 mutation, CAR-binding ablation (S408E, P409A) [29]; A20 peptide, NAVPNLRGDLQVLAQKVART; vp/mL, virus particles/mL.

Four Ad5-based (+/− CAR binding/A20 peptide) and two chimeric Ad5/kn48 (+/− A20 peptide) vectors were engineered by homologous AdZ recombineering [33]. Viruses were successfully produced to high titers in T-REx-293 cells (Figure 1A), and fiber integrity verified by Western blotting using anti-Ad fiber antibody 4D2 (Figure 1B). Sequence alignment of the knob domains revealed Ad48 knob to share three out of four key CAR-binding residues with Ad5, including the two residues central for ablation of CAR-binding (KO1) (Figure 1C). Incorporation of A20 peptide into the CD, HI or IJ loops of Ad5/kn48 was found to be incompatible with production of infectious virus (data not shown), while A20 insertion into the DG loop (Ad5/kn48.DG.A20) resulted in a functional virus. A panel of cell lines and two ovarian ascites-derived primary cultures were selected for *in vitro* and *ex vivo* transduction assays, based on their varying CAR and αvβ6 expression profiles in flow cytometry analysis (Figure 1D).

### A20 peptide increased transduction in αvβ6-positive cancer cells

**Figure 2:**
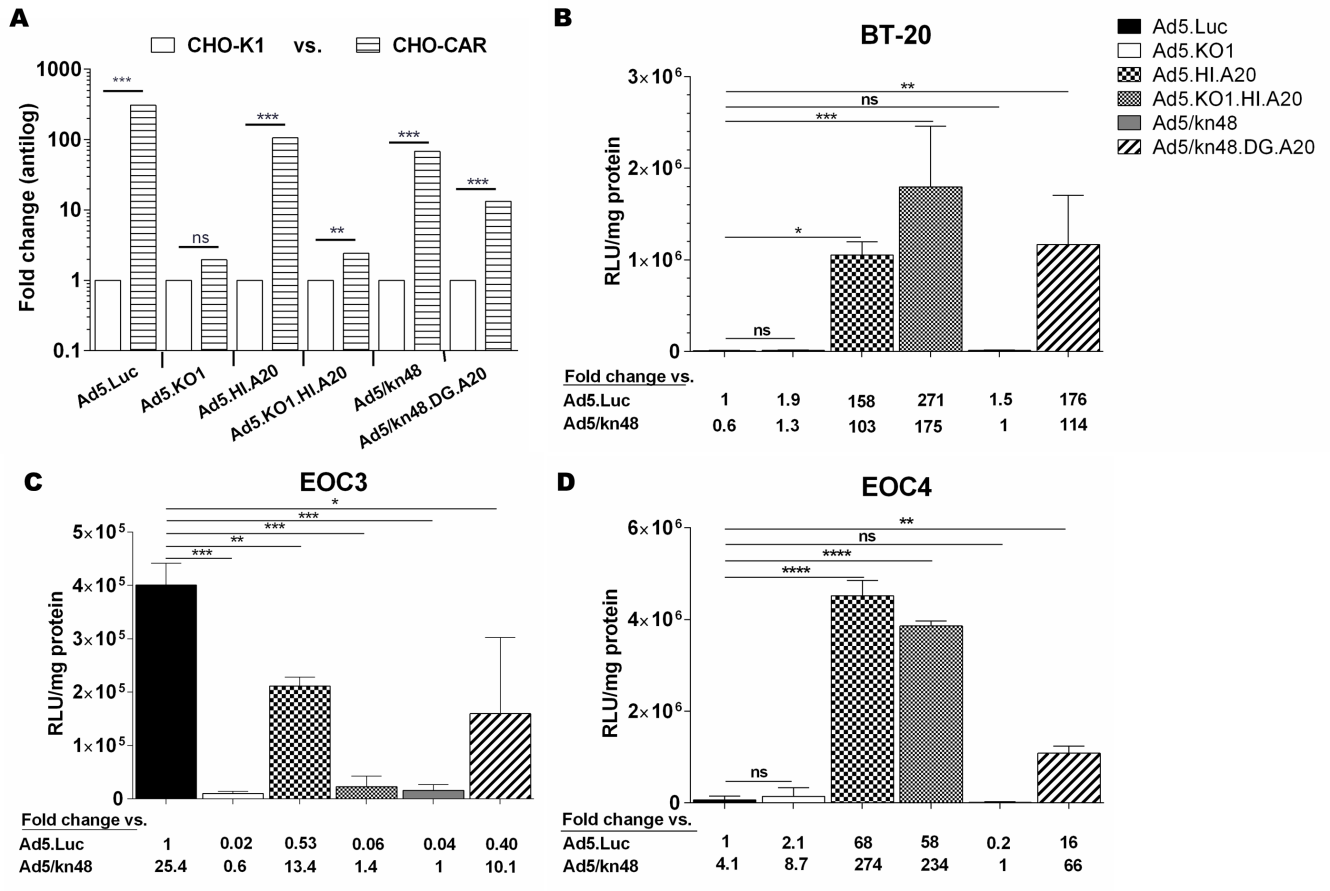
Vector transduction in αvβ6 integrin-expressing cancer cells **A.** Control cell lines: Chinese Hamster cells (CHO-K1 and CHO-CAR; n = 3); *p* values by two-tailed unpaired *t* test, **B.** Breast carcinoma (BT-20) cells in triplicate (n = 3), **C.** Ascites-derived epithelial ovarian cancer (EOC) cells from patient 3 in triplicate (n = 3) and **D.** EOC cells from patient 4 in duplicate (n = 2) were infected with 5000 virus particles/cell for 3 h, luciferase activity measured 48 h later and relative light units normalized to total cellular protein (RLU/mg). Data are representative of minimum of two independent experiments ± SD. Statistical significance shown as relative to Ad5.Luc using Dunnett's multiple comparisons test; adjusted *p*: ns > 0.05, * < 0.05, ** < 0.01, *** < 0.001, **** < 0.0001.

All CAR-binding viruses were shown to efficiently transduce CHO-CAR (CAR^high^/αvβ6^neg^) but not the control CHO-K1 (CAR^neg^) cells (*p* < 0.001), whilst KO1 mutation successfully ablated transduction (Figure 2A) as demonstrated previously [20]. These data strongly suggested the involvement of CAR receptor in the cellular transduction for the Ad48 fiber knob protein. BT-20 breast carcinoma cells (CAR^low^/αvβ6^high^) were used as a αvβ6-positive control cell line. Ad5/kn48-pseudotyping resulted in similar level of transduction to that seen with the unmodified Ad5.Luc in BT-20 cells (Figure 2B; *p* = 0.9978). Conversely, transduction of the A20-modified viruses Ad5.HI.A20, Ad5.KO1.HI.A20 and Ad5/kn48.DG.A20 increased by ∼ 160-, 270- and 180-fold relative to Ad5.Luc (Figure 2B; *p* = 0.0138, 0.0002 and 0.0068, respectively).

Variable transduction patterns were observed in freshly-isolated primary EOC cultures (Figure 2C-2D), consistent with their differing levels of receptor expression. The pattern seen in EOC3 cells (CAR^med^/αvβ6^med^) was similar to CAR^high^ CHO-CAR cells (Figure 2A), with significantly lower levels of transduction for all viruses as compared to Ad5.Luc (Figure 2C; *p* < 0.0001). Importantly, transduction in EOC4 cells (Figure 2D; CAR^med^/αvβ6^high^) was similar to the transduction pattern observed in BT-20 cells (Figure 2B), showing significant increase in transduction for all A20-modified vectors as compared to Ad5.Luc (*p* < 0.0001). A20 incorporation increased Ad5.HI.A20, Ad5.KO1.HI.A20 and Ad5/kn48.DG.A20 transduction in EOC4 cells by 68-, 58- and 16-fold as compared to Ad5.Luc (Figure 2D; *p* < 0.0001, < 0.0001 and 0.0041, respectively).

### Ad5/kn48 utilized CAR for cell entry

**Figure 3:**
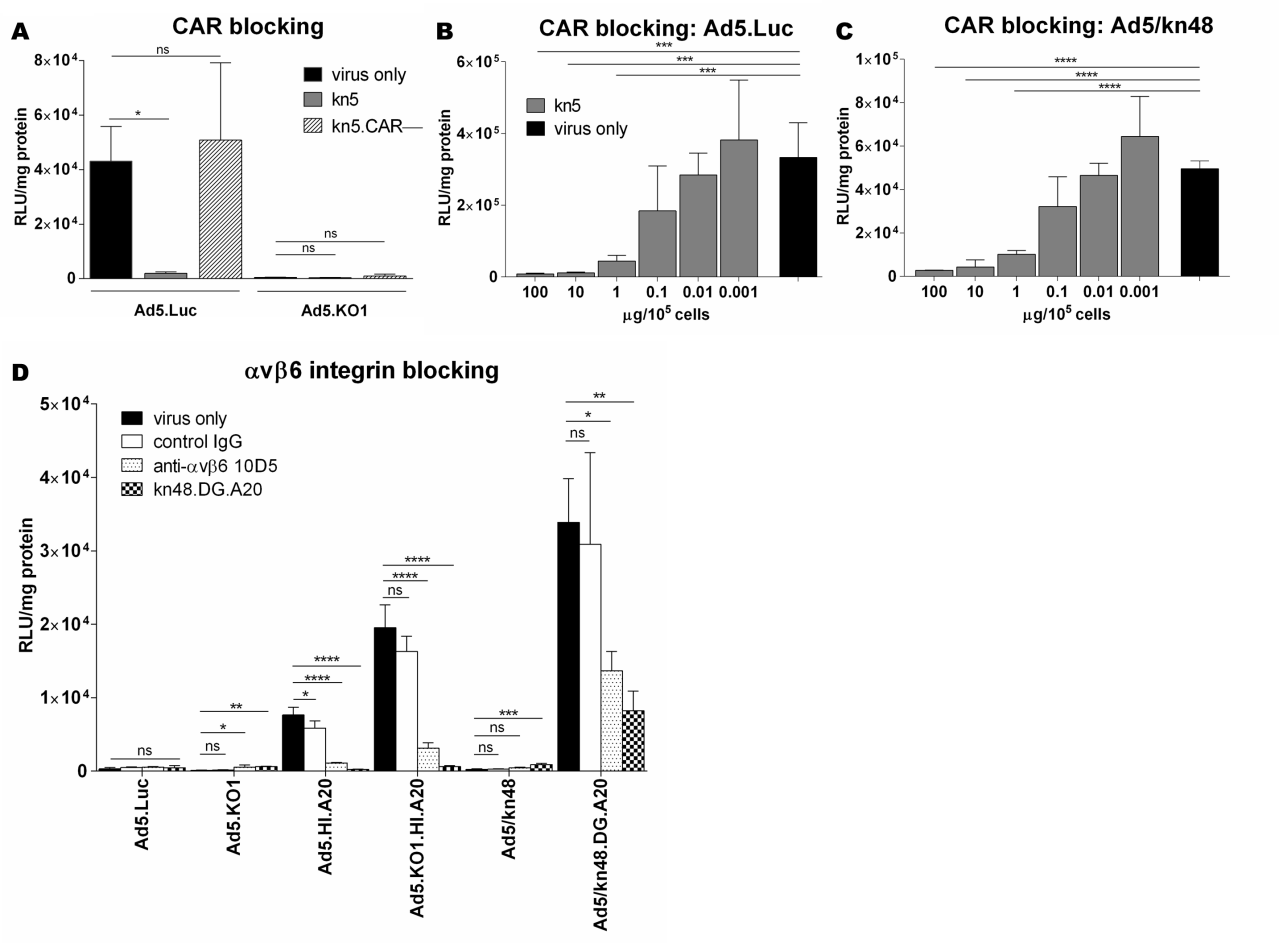
Evaluation of coxsackie and adenovirus receptor (CAR) and αvβ6 integrin receptor usage Cells were pre-blocked with soluble 6 x His-tagged Ad5 knob (kn5), Ad5 knob with ablated CAR-binding (kn5.CAR–), Ad48 knob with αvβ6-targeting peptide (A20) in DG loop (kn48.DG.A20) proteins (10 μg/10^5^ cells), normal mouse control IgG (10 μg/ml) or anti-αvβ6 antibody 10D5 (10 μg/ml) for 30 min on ice. Viruses were added at 5000 virus particles/cell for 1 h on ice, luciferase activity quantified 48 h later and relative light units normalized to total cellular protein (RLU/mg). **A.** CAR-binding competition inhibition assay in Chinese Hamster Ovary cells expressing CAR (CHO-CAR) in triplicate (n = 3). **B-C.** Dose-response CAR-binding competition inhibition assay in CHO-CAR cells, pre-blocking with kn5 protein at 100–0.001 μg/10^5^ cells in quadruplicate (n = 4). **D.** αvβ6 integrin competition inhibition assay in breast carcinoma (BT-20) cells. Data are representative of a minimum of two independent experiments in triplicate (n = 3), ± SD. Statistical significance shown as relative to ‘virus only’ conditions for each vector using Dunnett's multiple comparisons test; adjusted *p*: ns > 0.05, * < 0.05, ** < 0.01, *** < 0.001, **** < 0.0001.

Competition inhibition assays were performed in CHO-CAR (CAR^high^) cells to assess the receptor usage of the knob-pseudotyped viruses (Figure 3A-3C). Ad5.Luc was confirmed to utilize CAR for cell entry (Figure 3A), as transduction was inhibited by pre-blocking the CAR receptor with kn5 protein (*p* = 0.0353), while CAR-binding ablated control kn5.CAR– protein had no effect on transduction efficiency in CHO-CAR cells (*p* = 0.5646). No inhibition of Ad5.KO1 transduction by kn5 or kn5.CAR– (*p* = 0.9743 and 0.2342, respectively) was observed. Cell entry of Ad5.Luc and Ad5/kn48 via CAR was further assessed in dose-response assays. Significant reduction in Ad5.Luc transduction was observed (Figure 3B) at concentrations 100, 10 and 1 μg of kn5 protein/10^5^ cells (*p* = 0.0003, 0.0003 and 0.0010, respectively). Similarly efficient inhibition was achieved for Ad5/kn48 at equal concentrations of kn5 (Figure 3C; *p* < 0.0001 at all three doses). We further assessed the 50 % inhibitory concentration (IC_50_) of kn48 and found it to be ∼ 1000-fold higher than that of kn5 (Figure S2A), suggesting an affinity to CAR lower than kn5.

### A20-modified virus transduction was αvβ6 integrin-mediated

Successful binding to αvβ6 integrin was confirmed for Ad5.HI.A20, Ad5.KO1.HI.A20 and Ad5/kn48.DG.A20 vectors in competition inhibition assays in BT-20 (αvβ6^high^) cells (Figure 3D). Ad5.Luc transduction was not significantly inhibited by any of the blocking agents as compared to ‘virus only’ conditions (*p* = 0.4806). Anti-αvβ6 antibody and kn48.DG.A20 significantly inhibited both Ad5.HI.A20 and Ad5. KO1.HI.A20 transduction (*p* < 0.0001 for all). Ad5/kn48 vector was inhibited by kn48.DG.A20 protein only (*p* = 0.0001). Importantly, Ad5/kn48.DG.A20 transduction was significantly inhibited by both anti-αvβ6 antibody and kn48.DG.A20 protein (*p* = 0.0213 and 0.0059, respectively).

### Ad5/kn48 did not use CD46 as its primary receptor

**Figure 4:**
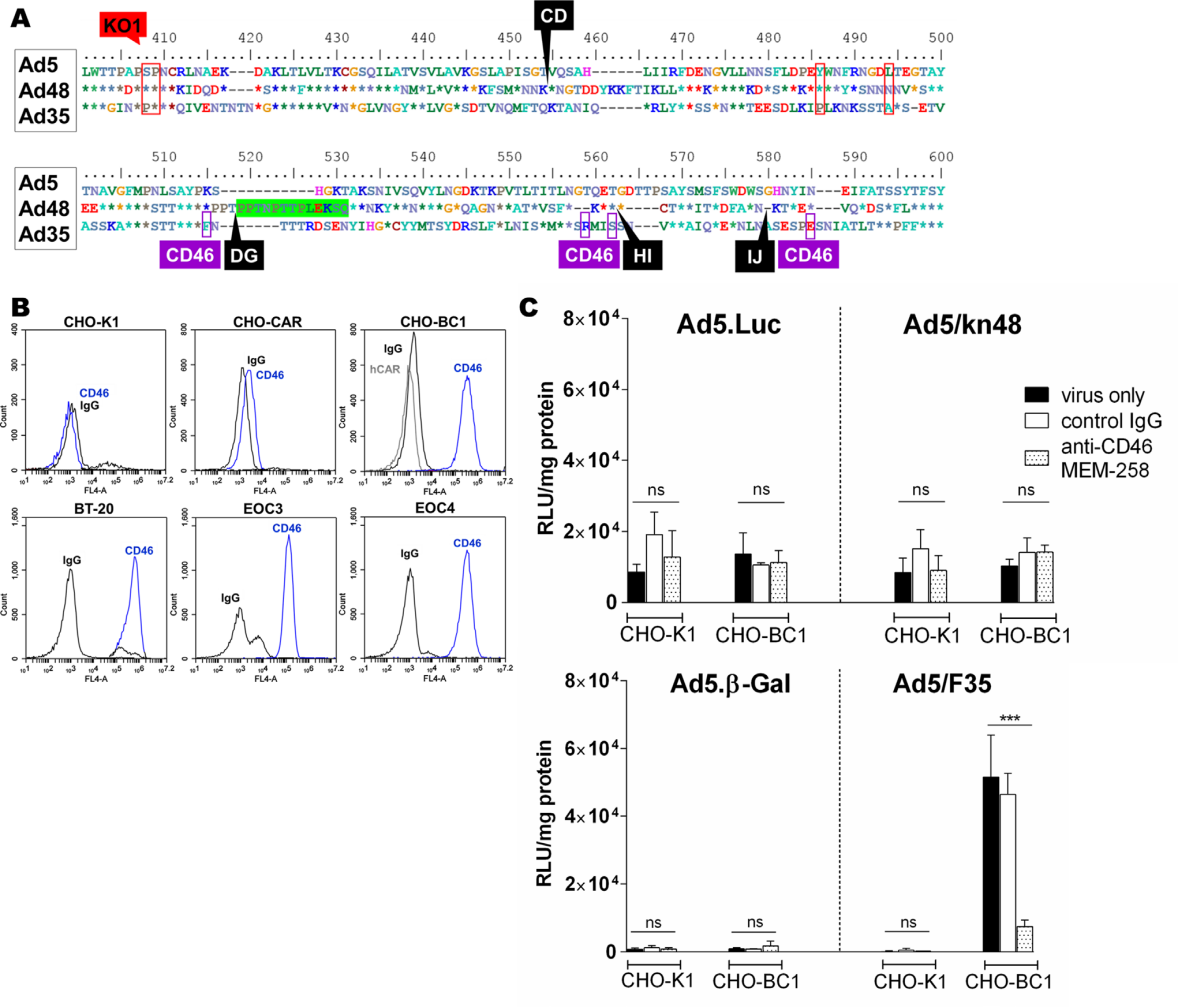
Evaluation of CD46 receptor usage **A.** Ad5, Ad48 and Ad35 fiber knob alignment: red boxes, coxsackie and adenovirus receptor (CAR)-binding sites [3]; green box, 13-aa deletion; black labels, A20 insertion sites/knob loops; purple boxes, CD46-binding sites [42]. **B.** CD46 receptor expression was assessed in Chinese Hamster Ovary (CHO-K1), CHO cells expressing CAR (CHO-CAR), CHO expressing CD46 (CHO-BC1), breast carcinoma (BT-20) cell lines and ascites-derived primary ovarian epithelial cancer (EOC) cells from a donor 3 (EOC3) and donor 4 (EOC4) by flow cytometry (normal mouse IgG, sc-2025; anti-CAR, Rcmb; anti-CD46, MEM-258). Data was analyzed on BD Accuri C6 software. Competition inhibition assay in CHO-K1 and CHO-BC1 cells using **C.** luciferase (Luc) top panel and β-Gal-expressing viruses bottom panel. Cells were pre-incubated with normal mouse control IgG (10 μg/ml) or anti-CD46 clone MEM-258 (10 μg/ml) for 30 min on ice and infected with 5000 viral particles/cell for 1 h on ice. Luciferase/β-galactosidase (β-Gal) expression was measured 48 h post-infection and relative light units were normalized to total cellular protein (RLU/mg). Data shown are representative of a minimum of two independent experiments in triplicate (n = 3), ± SD. Statistical significance shown as relative to ‘virus only’ conditions using Dunnett's multiple comparisons test; adjusted *p*: ns > 0.05, * < 0.05, ** < 0.01, *** < 0.001.

Species D Ads have previously been suggested to utilize CD46 as their primary entry receptor [12, 41]. However, fiber knob alignment showed CD46-binding residues [42] to be absent in the Ad48 knob domain (Figure 4A). As BT-20 cells and the primary EOC cells express high levels of CD46 (Figure 4B), competition inhibition assays were performed in CHO-BC1 (CAR^neg^/CD46^high^) cells using relevant CD46-utilizing control viruses to further assess the potential involvement of CD46 in Ad48 cell entry (Figure 4C). As expected, there were no differences in cell transduction in the CD46^neg^ control cell line CHO-K1 for Ad5.Luc or Ad5/kn48 as compared to ‘virus only’ conditions (Fig. 4C, top panel; (*p* = 0.1568 and 0.2231, respectively). Similarly, anti-CD46 antibody did not inhibit transduction of these viruses in CHO-BC1 cells (*p* = 0.6382 and 0.2307, respectively), and no inhibition of CD46 detection was observed by flow cytometry following pre-incubation of cells with either kn48 or the CAR-binding kn5 protein (Figure S2B). Conversely, CD46-mediated entry of Ad5/F35 control vector [35, 36] into CHO-BC1 cells was completely inhibited by anti-CD46 antibody (Fig. 4C, bottom panel; (*p* = 0.001), with no significant inhibition observed in CHO-K1 cells (*p* = 0.3883).

### A20 insertion enabled efficient transduction in the presence of neutralizing ovarian ascites

**Figure 5:**
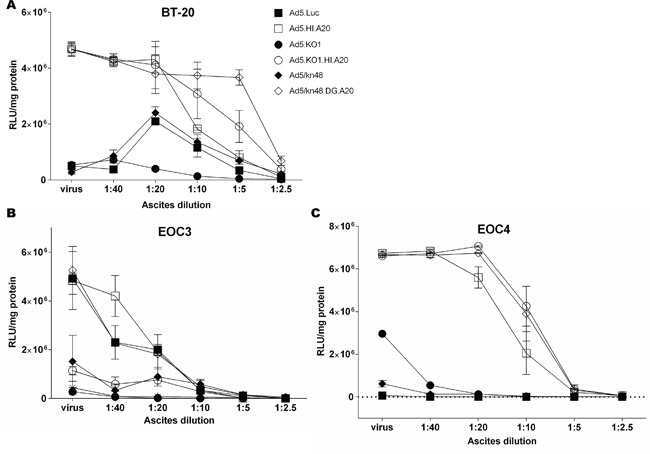
Vector neutralization by ovarian ascites **A.** Breast cancer (BT-20) cells, **B.** ascites-derived primary ovarian epithelial ovarian cancer (EOC) cells from donors 3 and **C.** 4 were incubated with 2-fold dilutions of highly-neutralizing cell-free ascites (Fig. S3, donor 1) for 30 min at 37°C, and infected at 200 000 virus particles/cell for 2 h at 37°C. Luciferase activity was measured 48 h later and relative light units normalized to total cellular protein (RLU/mg). Data are representative of a minimum of two independent experiments in duplicate (n = 2). ‘virus’, virus only in ascites- and serum-free conditions.

We [20] and others [17–19] have previously demonstrated that ascites contains high levels of nAbs that efficiently neutralize Ad5 vectors. To evaluate the effect of knob-pseudotyping and A20 insertion on protection from pre-existing nAbs, transduction assays were performed in the presence of increasing concentrations of highly neutralizing cell-free ascites. Ascites from donor 1 was the most efficient at neutralizing Ad5.Luc (Figure S3) and was used in all neutralization assays. Efficient transduction was maintained for all αvβ6-targeted vectors in BT-20 cells (αvβ6^high^) in the neutralizing conditions, in contrast with the vectors with no available entry receptor (Figure 5A; Table S2A). To demonstrate the clinical scenario, virus transduction was assessed in primary *ex vivo* cultures EOC3 (αvβ6^med^) and EOC4 (αvβ6^high^) (Figure 5B-5C). In EOC3 cells, Ad5.Luc, Ad5.HI.A20 and Ad5/kn48.DG.A20 were the only vectors to resist neutralization in the presence of 2.5 and 5 % ascites, while all vectors were completely neutralized at higher ascites concentrations (Figure 5B; Table S2B). Conversely, all αvβ6-targeted vectors transduced EOC4 cells at a greatly increased efficiency compared to Ad5.Luc (Table S2C) at up to 20 % ascites (Figure 5C), showing transduction patterns similar to that in the αvβ6^high^ BT-20 cells (Figure 5A).

## DISCUSSION

Recent positive results from the OPTiM phase III clinical trial of talimogene laherparepvec (T-VEC) [43] demonstrate that adjunctive oncolytic virotherapies are beginning to gain ground among anti-cancer therapies. The oncolytic herpes simplex virus type 1-based T-VEC showed improved durable response rate (16 % vs. 2 %) compared to the control arm (granulocyte-macrophage colony-stimulating factor, GM-CSF), and an improved 33.4 % 5-year overall survival [44]. T-VEC was licensed by the U.S. FDA in October 2015 and by EMA in December 2015 as the first oncolytic immunotherapeutic (Imlygic) in Europe for the treatment of metastatic melanoma [45]. Over a decade ago, the first oncolytic, conditionally-replicative Ad (CRAd) ‘H101′ was approved in China for combination therapies of head and neck carcinomas [46], and is now clinically available. Phase I/II clinical trials are currently ongoing for the treatment of advanced colorectal and other solid tumors with enadenotucirev (formerly ColoAd1), a chimeric Ad11p/Ad3 vector that has shown high selectivity, potent cancer-killing [47] and protection from anti-Ad humoral immunity [48].

Efficient systemic delivery of virotherapies is the long-term goal for metastatic cancer treatment but remains problematic due to toxicity-inducing 'off-target' binding (reviewed in [2]). KO1 mutation [20, 29] presents a feasible strategy for abolishing binding to CAR that is ubiquitously expressed in healthy tissues but suppressed on various tumor types [9, 10]. Localized, i.p. treatment of ovarian cancer metastases would circumvent systemic interactions but therapeutic efficacy of Ad5-based vectors is severely hampered by high prevalence of anti-Ad5 nAbs in the ascites [17–20]. Ascites-resident nAbs seem to be primarily anti-fiber [17], which is why fiber modification by heterologous peptide incorporation and/or pseudotyping may protect from neutralization (reviewed in [49]). Tropism-modified Ad5 vectors with peptide-engineered fibers have previously showed improved *in vitro* transduction in the presence of neutralizing ascites [17, 20]. An alternative strategy to ablate native Ad5 tropisms is via chemical modification the capsid with hydrophilic polymers such as poly(*N*-(2-hydroxypropyl) methacrylamide) (pHPMA). Using this approach it has been possible to improve tumor-selectivity in i.p. ovarian cancer models via subsequent attachment of epidermal growth factor receptor (EGFR) [15, 50]. However, clinical efficacy of chemically-coated Ads may be limited, since chemical modification and targeting ligands are not heritable by the virus progeny.

Species D Ads present an appealing alternative to Ad5-based vectors due to their low seroprevalence [11, 12], and alternative tissue tropisms [51–54]. Intriguingly, Ad48 has shown resistance to pre-existing anti-Ad5 nAbs [55], greater replication efficiency and oncolytic potency in B cell cancers compared to Ad5 [56]. In this study, we have shown that three out of four amino acids central for Ad5 CAR binding [57] are conserved in the Ad48 knob domain (Figure 1C), thus supporting the finding that Ad5/kn48 vector utilizes CAR for cell entry (Figure 3C). Future modifications may therefore include the introduction of KO1 mutation to abolish CAR-binding. Species D Ads have been previously suggested to utilize CD46 as their primary entry receptor [12, 41, 54]. This observation was not supported here as CD46 was found to be non-essential for cell entry (Figure 4C and S2B). However the lower affinity of kn48 for CAR (Figure S2A) could imply the involvement of additional co-receptors that remain to be determined in prospective studies.

Poor tumor-specificity remains one of the key hurdles in Ad vector design. This may be overcome by genetic insertion of peptide ligands for targeting tumor cell-specific receptors. αvβ6 integrin is undetectable in normal adult tissues [21, 22] but up-regulated in many cancer types, such as ovarian [23], breast [58], colon [59], prostate cancer [60] and cancer of the oral cavity [61], as well as squamous carcinomas of the cervix, skin, esophagus, and head and neck [24]. Its cancer-specific expression and established role as a prognostic indicator [22, 25, 26] make it an appealing target for anti-cancer vectors. A20, originally derived from VP1 capsid protein of FMDV [27], natively binds αvβ6 integrin with high affinity, and has previously been utilized for Ad5 tumor-targeting *in vivo* [28].

A20 peptide was inserted into the Ad5 fiber knob HI loop [28], and CD, DG, HI and IJ loops within Ad5/kn48 by homologous recombineering [33]. Predictive 3D models [39] showed A20 in Ad48 DG and HI loops in a favorably-exposed conformation for receptor binding (Figure S1). A20 insertion into DG and HI loops of recombinant kn48 protein has previously been shown to impair fiber trimerization, suggesting these sites to be suboptimal [34]. Conversely, in this study DG was the only loop that tolerated A20 insertion in combination with a 13-aa deletion, allowing functional Ad5/kn48 virus production (Figure 1A-1B). A20 behaved differently in the context of the whole virion, likely due to unpredicted steric hindrance and conformational changes. Ad5 fiber knob is well-characterized for re-targeting strategies [62–64], with knob loops HI [65], CD and IJ [66] known to tolerate insertion of targeting peptide moieties without compromised viral functionality. However, direct comparison of Ad5 and Ad48 is problematic as they belong to different subgroups (48 % fiber knob homology).

Incorporation of A20 rendered αvβ6^high^ virus-resistant cancer cell lines and αvβ6^high^ primary EOC *ex vivo* cultures susceptible to transduction and resulted in improved re-targeting relative to the CAR-utilizing parent vector Ad5.Luc (Figure 2). Competition inhibition assays utilizing function-blocking agents demonstrated that cell transduction was indeed CAR- and/or αvβ6 integrin-mediated (Figure 3). The observed differences in transduction efficiency between the primary cultures EOC3 and EOC4 (Figure 2) are consistent with well-recognized inter-patient heterogeneity of tumors. An estimated 33 % of ovarian cancers express αvβ6 integrin [24], and therefore stratification of patients may be required for a personalized virotherapy approach.

Ascites-resident anti-Ad5 nAbs [17–20] can severely hinder i.p. ovarian cancer virotherapies. Intriguingly, all αvβ6 integrin-targeted vectors demonstrated efficient transduction in αvβ6^high^ breast cancer cell line and primary αvβ6^high^ EOC *ex vivo* cultures in the presence of highly neutralizing clinical ascitic fluid (Figure 5A, 5C). The protective effect was less pronounced in primary EOC3 cells (Figure 5B), likely due to lower levels of cellular target receptors. A20 not only enabled cell transduction via a non-native, cancer-specific pathway but may also have contributed to the occlusion of neutralizing epitopes within the fiber knob. Adenoviral cancer-targeting via αvβ6 integrin merits further investigation and manipulation for oncolytic purposes. Local administration of adjunctive virotherapies has vast potential for fighting aggressive, relapsed forms of ovarian cancer.

## MATERIALS AND METHODS

### Cell lines, primary cell culture and flow cytometry

All cell lines were originally obtained from American Type Culture Collection (ATCC). T-REx-293 Human Embryonic Kidney cells were grown in Dulbecco's Modified Eagle's Medium (DMEM), A549 lung carcinoma cells in Roswell Park Memorial Institute (RPMI) 1640, BT-20 breast carcinoma cells in Eagle's Minimum Essential Medium (EMEM, α modification), Chinese Hamster Ovary cells (CHO-K1), CHO cells expressing CAR (CHO-CAR) and CHO cells expressing CD46 receptor isoform BC1 [32] (CHO-BC1) in DMEM: Nutrient Mixture F12. All media were supplemented with 4 mM L-Glutamine, 100 U/mL penicillin, 100 μg/mL streptomycin and 10 % fetal calf serum (except 20 % for BT-20), and cells grown at 37^°^C in a humidified atmosphere with 5 % CO_2_. All reagents were purchased from Gibco (Paisley, UK) or Sigma Aldrich (Gillingham, UK).

Permission for the collection and cultivation of primary EOC cells from ascites was granted through a Wales Cancer Bank application for biomaterials (reference WCB 14/004). All patients gave written informed consent prior to collection. Samples from two donors undergoing treatment at Velindre Cancer Centre, Cardiff were anonymized – EOC3 was from a patient with no prior history of chemotherapy and EOC4 from a patient with relapsed, platinum-resistant disease. Cells were collected by centrifugation of 500 mL ascites, processed and sub-cultured as described previously [20].

Receptor expression profiles were assessed by flow cytometry. Cells were incubated with normal mouse IgG control sc-2025 (1:200; Santa Cruz Biotechnology, Heidelberg, Germany), mouse anti-CAR clone Rcmb (1:500; Millipore, Watford, UK), mouse anti-αvβ6 clone 10D5 (1:100; Millipore, Middlesex, UK) or mouse anti-CD46 clone MEM-258 (1:100; Abcam, Cambridge, UK) in triplicate. 10 000–20 000 events were recorded in channel FL-4 and data assessed on BD Accuri C6 (BD Biosciences) flow cytometer. The flow cytometry data were analyzed using BD Accuri C6 software version 1.0.264.21 (Becton Dickinson, USA) as described previously [20].

### Recombinant adenoviruses

Pseudotyped Ad5/Ad48 vectors were generated by AdZ homologous recombineering as described previously [33]. Viral genomes were based on luciferase (Luc)-expressing replication-deficient (ΔE1/ΔE3) (Ad5.Luc) and introduced modifications were verified by sequencing [20]. A20 insertion sites in Ad5 [28] and Ad48 knob loops [34] were selected based on conformational compatibility for receptor binding (Figure S1). Ad48 knob sequences were PCR-amplified from previously generated pQE30knob48 plasmids [34] (primers, Table S1) and inserted into Ad5.Luc fiber knob (Ad5 fiber sequence before TLW hinge region (aa 400–402), Ad48 fiber afterwards). To generate Ad5.HI.A20, A20 sequence was amplified from the previously engineered pQE30knob48.DGΔ13aa.A20 plasmid [34] by PCR and inserted into the Ad5.Luc fiber knob HI loop (aa 546/547). The Ad5/kn48.A20 has a 13-aa deletion in the fiber knob prior to the site of A20 insertion [34] (aa 297–311) (Figure 1C). CAR-binding ablating mutation KO1 [29] was introduced into the fiber knob AB loop (S408E, P409A). All vectors were produced, purified and characterized as described previously [20]. The β-galactosidase (β-Gal) expressing control vectors Ad5.β-Gal and Ad5 with HAdV-B35 fiber (Ad5/F35) [35, 36] were a kind gift from Prof. Andrew Baker, University of Glasgow, UK.

### Transduction, neutralization and competition inhibition assays

Transduction, competition inhibition and neutralization assays were performed using the Luciferase Assay System kit (Promega UK Ltd, Southampton, UK) for the Luc-expressing viruses as described previously [20]. For quantification of the β-Gal expressing control virus Ad5.β-Gal and Ad5/F35 transduction, β-Gal expression was measured using Galacto-Light Plus System according to manufacturer's instructions (Applied Biosystems, Bedford, Massachusetts, USA). Luminescence was measured on a multimode plate reader (FLUOstar Omega, BMG Labtech, Aylesbury, UK). Values were expressed as relative light units (RLU) and normalized for total cellular protein (RLU/mg), as described previously [20].

For CAR competition inhibition assays, soluble, recombinant 6 x His-tagged knob proteins were produced as described previously [28, 34]; wild type Ad5 knob (kn5), Ad5 knob with a CAR-binding ablation mutation Y477A in the DE loop [37] (kn5.CAR–), wild type Ad48 knob (kn48) and Ad48 knob with a 13-aa deletion and A20 peptide in the DG loop (kn48.DG.A20). Cells were pre-incubated with the soluble knob proteins (10 μg/10^5^ cells), function-blocking anti-αvβ6 antibody clone 10D5 (10 μg/ml), function-blocking anti-CD46 clone MEM-258 (10 μg/ml) or normal anti-mouse control IgG (10 μg/ml) for 30 min on ice and viruses added for further 1 h on ice. In dose-response assays, cells were pre-blocked with 0.001–100 μg of kn5 protein/10^5^ cells. Flow cytometry was used to determine the ability of recombinant kn5 and kn48 proteins (0.0001–100 μg/10^5^ cells) to bind and competitively inhibit anti-CAR (RmcB) (Figure S2A) and anti-CD46 (MEM-258) antibody binding (Figure S2B) as described previously [28, 34]. Cell-free ascitic fluids from five donors were tested on A549 lung carcinoma cells (CAR^high^) for selection of the most neutralizing sample (Figure S3). This fluid (donor 1) was used in all neutralization assays.

### Sequence alignments and predictive structural modelling

Ad5 (GenPept: AAP31231.1), Ad48 (GenPept: ABO61306.1) and Ad35 (GenPept: AP_000601.1) fiber sequences were aligned in BioEdit [38] version 7.2.5 using the ClustalW Multiple Alignment command. A20-modified knob domains were modelled in predictive online 3D modelling software SWISS-MODEL [39] (Biozentrum, Basel, Switzerland) on automated mode and saved as Protein Data Bank (PDB) files for further editing. Ad5 knob models were based on PDB structure 1KNB and Ad48 knobs based on the closest homology (64 %) HAdV-D19p structure (PDB ID: 1UXB) as crystallization data for Ad48 is not available. Molecular graphics and analyses were performed using the UCSF Chimera package [40] version 1.10.2 (Resource for Biocomputing, Visualization, and Informatics; University of California, San Francisco, USA).

### Statistical analyses

Two-tailed unpaired *t* test was used for analysis in Figure 2A. One-way ANOVA with Dunnett's multiple comparisons post hoc test was used in all other assays. All analyses and graphs were created in GraphPad Prism version 6.03 (GraphPad Software Inc., La Jolla, CA, USA).

## SUPPLEMENTARY FIGURES AND TABLES






