# Supplementary material for: Pseudotyped αvβ6 integrin-targeted adenovirus vectors for ovarian cancer therapies
Source: Oncotarget. 2016 Apr 1;7(19):27926–37. doi: 10.18632/oncotarget.8545 (PMC5053699; doi:10.18632/oncotarget.8545)
Supplement: Supplementary file 1 [file oncotarget-07-27926-s001.pdf]

SUPPLEMENTARY FIGURES AND TABLES

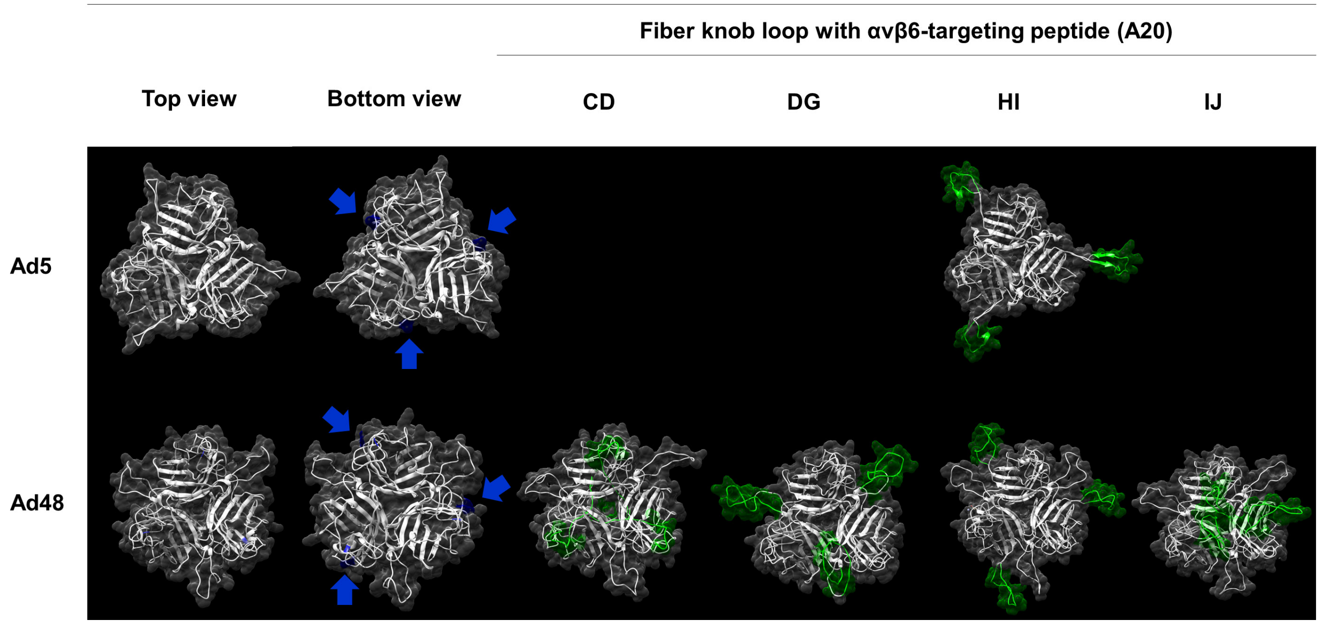

Supplementary Figure S1: Predictive structural modelling of native and αvβ6-targeted Ad5 and Ad48 fiber knob domains. Ad5 and Ad48 knobs (GenPept: AAP31231.1 & ABO61306.1) with coxsackie and adenovirus receptor (CAR)-binding site [3] in blue and A20 peptide (NAVPNLRGDLQVLAQKVART) insertion in green. Structures were modelled in SWISS-MODEL software [39], and molecular graphics and analyses were performed with the UCSF Chimera package [40] (University of California, San Francisco, USA) version 1.10.2. Ad5 knob models were based on Ad5 knob structure (PDB ID: 1KNB) and Ad48 knob models on Ad19p structure (PDB ID: 1UXB).

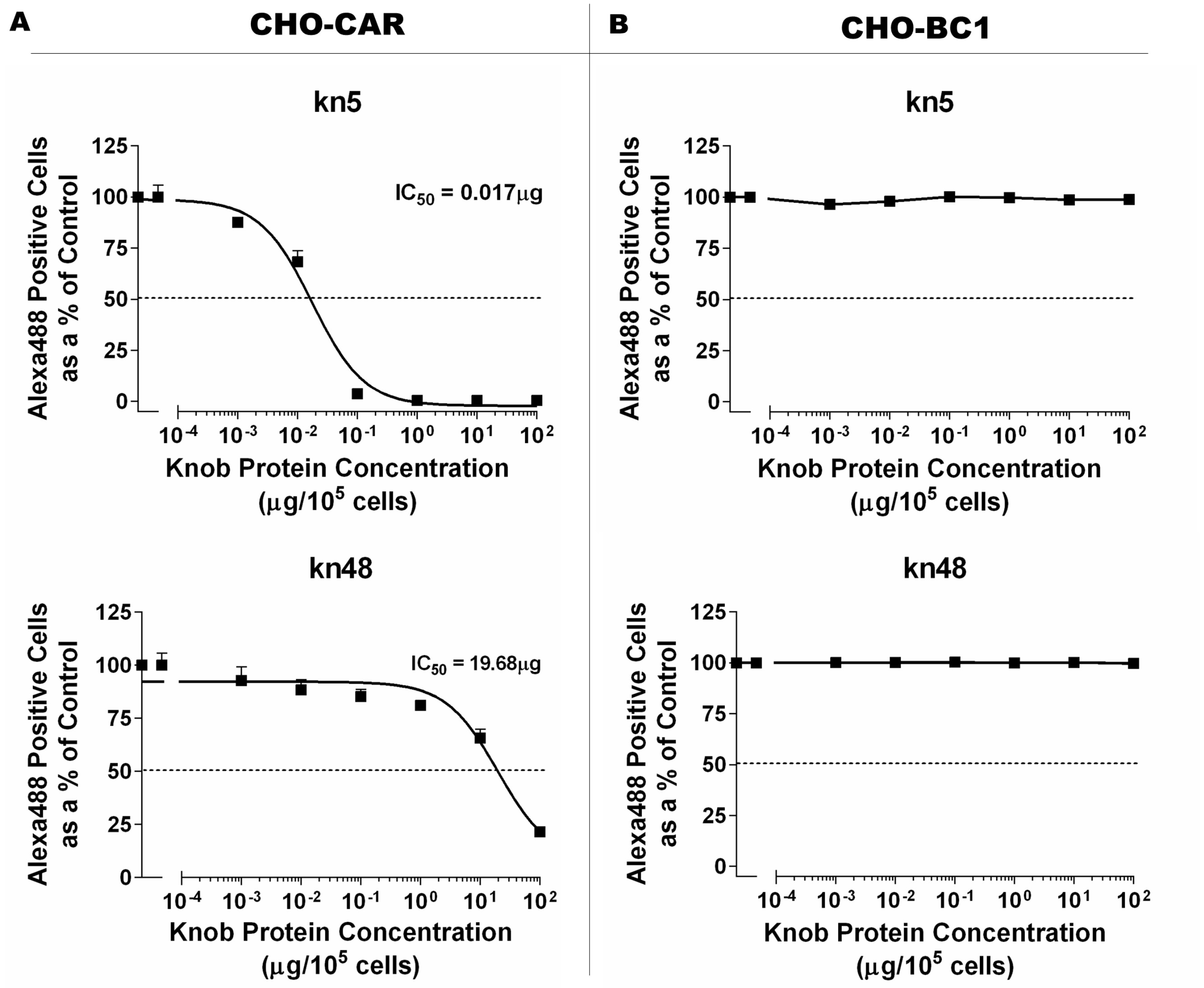

Supplementary Figure S2: Determination of 50 % inhibitory concentration (IC50) of recombinant Ad5 knob (kn5) and Ad48 knob (kn48) proteins. CHO-CAR and CHO-BC1 cells were incubated with increasing concentrations of kn5 or kn48 protein (0.0001–100 µg/10^5 cells) for 1 h on ice. Control cells were incubated with serum-free medium. **A.** An anti-CAR antibody (RmcB) or **B.** anti-CD46 antibody (MEM-258) was added to all samples and binding detected using anti-mouse Alexa488 by flow cytometry. Fluorescence of control cells (no knob pre-treatment) are expressed as 100 % and all treated samples as a percentage of this. Data show the mean ±SD and are representative of two independent repeat experiments in triplicate (n = 3).

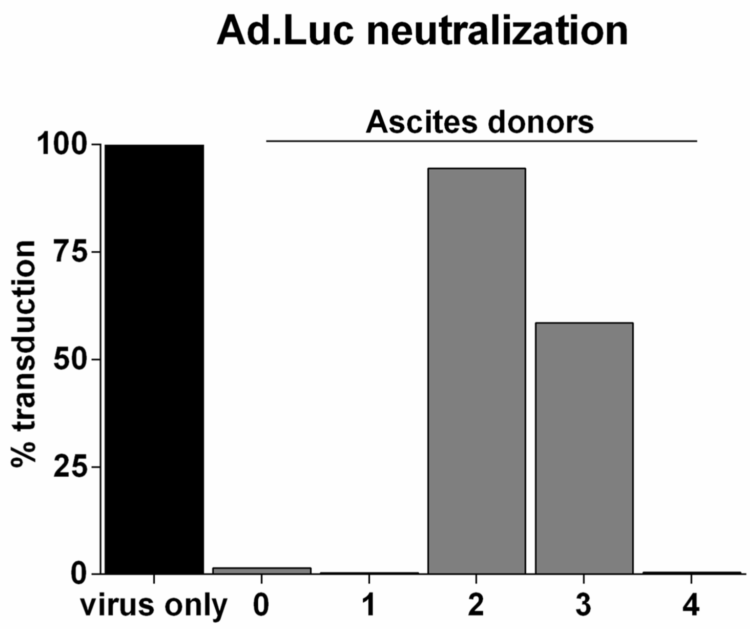

Neutralization (%)	98.5	99.6	5.5	41.5	99.5
Fold change vs. donor 1	4.2	1	265	164	1.4

Supplementary Figure S3: Neutralization of Ad5.Luc by ovarian ascites. 2.5 % cell-free ascites fluids from five donors were assessed for Ad5.Luc neutralization in A549 lung carcinoma cells (CAR^{high}). Cells were infected with 5000 viral particles/cell in duplicate (n = 2), luciferase expression quantified 48 h later and relative light units normalized to total cellular protein (RLU/mg). Values expressed as % of 'virus only' (ascites-free) conditions. Sample with highest neutralization (donor 1) was chosen for subsequent neutralization assays.

Supplementary Table S1: Primers for Ad5/Ad48 fiber pseudotyping and A20 peptide insertion. *F*, forward primer; *R*, reverse primer; seq, sequencing primers used for verification of successful mutations introduced by recombineering.

See Supplementary File 1

Supplementary Table S2: Fold change in vector transduction in the presence of neutralizing ascites. Fold change in luciferase expression when raw values (relative light units, RLU/mg) are compared to Ad5.Luc in the same conditions in the presence of ascending 2-fold serial dilutions of cell-free ascites in (A) breast carcinoma (BT-20) cell line, (B) primary epithelial ovarian cancer (EOC) cells from donor 3 and C) EOC cells from donor 4. Values are based on the assay described in Fig. 5.

See Supplementary File 2
