# Supplementary material for: Pseudotyped αvβ6 integrin-targeted adenovirus vectors for ovarian cancer therapies
Source: Oncotarget. 2016 Apr 1;7(19):27926–37. doi: 10.18632/oncotarget.8545 (PMC5053699; doi:10.18632/oncotarget.8545)
Supplement: Supplementary file 2 [file oncotarget-07-27926-s002.pdf]

Table S1

Primer	Sequence
Cass in Ad5 fiber *F*	GTTCCTAAACTAGGAACTGGCCTTAGTTTTGACAGCACAGGTGCCATTACAGTAGGAAACAAAAATAATGATAAGCTAC CTGTGACGGAAGATCACTTCG
Cass in Ad5 fiber *R*	CTACTGAATGAAAAATGACTTGAAATTTTCTGCAATTGAAAAATAAACACGTTGAAACATAACACAAACGATTCTCTGAG GTTCTTATGGCTCTTG
Ad48 fragment *F*	GTTCCTAAACTAGGAACTGGCCTTAGTTTTGACAGCACAGGTGCCATTACAGTAGGAAACAAAAATAATGATAAGCTA
Ad48 fragment *R*	TACTGAATGAAAAATGACTTGAAATTTTCTGCAATTGAAAAATAAACACGTTGAAACATAACACAAACGATTCTTTATTCT TGGGCAATATAGGAGAAAG
A20 into Ad5 HI *F*	TCAGTCAAGTTTACTTAAACGGAGACAAAACTAAACCTGTAACACTAACCATTACACTAAACGGTACACAGGAAACAA A TGCTGTGCCCAACTTGAGAG
A20 into Ad5 HI *R*	CAAATATTTCATTAATGTAGTTGTGGCCAGACCAGTCCCATGAAAATGACATAGAGTATGCACTTGGAGTTGTGTCTCC CGTCCGTGCCACCTTTTGAG
A20 into HI seq *F*	TCAGTCAAGTTTACTTAAACGGAGACAAAACTAAACCTGTAACACTAACCATTACACTAAACGGTACACAGGAAACAA ATGCTGTGCCCAACTTGAGAG
A20 into HI seq *R*	CAAATATTTCATTAATGTAGTTGTGGCCAGACCAGTCCCATGAAAATGACATAGAGTATGCACTTGGAGTTGTGTCTCC CGTCCGTGCCACCTTTTGAG
Ad48 fiber seq *F*	CACTGCCAAGGGGTTGATG
Ad48 fiber seq *R*	CCAGCCGGGGAGAAAGG
