# Supplementary material for: Pseudotyped αvβ6 integrin-targeted adenovirus vectors for ovarian cancer therapies
Source: Oncotarget. 2016 Apr 1;7(19):27926–37. doi: 10.18632/oncotarget.8545 (PMC5053699; doi:10.18632/oncotarget.8545)
Supplement: Supplementary file 3 [file oncotarget-07-27926-s003.pdf]

Table S2

A

Virus	1:2.5	1:5	1:10	1:20	1:40	virus only
Ad5.Luc	1.0	1.0	1.0	1.0	1.0	1.0
Ad5.KO1	0.6	0.1	0.1	0.2	1.9	1.1
Ad5.HI.A20	2.3	2.3	1.6	2.0	11.1	9.2
Ad5.KO1.HI.A20	8.2	5.6	2.7	2.0	11.4	9.2
Ad5/kn48	4.6	2.0	1.2	1.1	2.3	0.6
Ad5/kn48.DG.A20	14.5	10.7	3.2	1.8	11.3	9.2

B

Virus	1:2.5	1:5	1:10	1:20	1:40	virus only
Ad5.Luc	1.0	1.0	1.0	1.0	1.0	1.0
Ad5.KO1	0.2	0.01	0.02	0.01	0.03	0.06
Ad5.HI.A20	0.4	0.1	1.0	1.0	1.8	1.0
Ad5.KO1.HI.A20	4.2	0.2	0.8	0.4	0.3	0.2
Ad5/kn48	14.7	1.4	1.7	0.4	0.1	0.3
Ad5/kn48.DG.A20	3.0	1.3	1.5	0.9	1.0	1.0

C

Virus	1:2.5	1:5	1:10	1:20	1:40	virus only
Ad5.Luc	1.0	1.0	1.0	1.0	1.0	1.0
Ad5.KO1	1.6	1.8	3.3	17.6	70.1	45.8
Ad5.HI.A20	23.1	59.3	369	753	876	104
Ad5.KO1.HI.A20	23.5	98.8	765	950	860	102
Ad5/kn48	3.5	4.0	6.0	17.4	16.9	9.6
Ad5/kn48.DG.A20	21.5	88.5	701	906	851	103
